# Media Internalized Pressure and Restrained Eating Behavior in College Students: The Multiple Mediating Effects of Body Esteem and Social Physique Anxiety

**DOI:** 10.3389/fpsyg.2022.887124

**Published:** 2022-06-15

**Authors:** Tiantian Fu, Jun Wang, Shanshan Xu, Jinrong Yu, Guoxiao Sun

**Affiliations:** School Physical Education, Shandong University, Jinan, China

**Keywords:** media internalized pressure, restrained eating behavior, body esteem, college students, social physique anxiety

## Abstract

**Background:**

Restrained eating behavior has become the norm in college students' lives, and considering the harm it causes to college students, it is necessary to explore the factors associated with it. The aim of this study was to explore the association between media internalized pressure, body esteem, social physique anxiety, and restrained eating behavior.

**Methods:**

The participants in this study were 1,032 Chinese college students (439 males and 593 females) and had a mean age of 20.22 years (SD = 1.277). They completed the Sociocultural Attitudes Toward Appearance Questionnaire-3, Body Esteem Scale (BES), Social Physique Anxiety Scale (SPAS), and Dutch Eating Behavior Questionnaire (DEBQ).

**Results:**

The results showed that media internalized pressure was significantly and positively associated with college students' restrained eating behavior, that body esteem and social physique anxiety played a mediating role between media internalized pressure and restrained eating behavior, respectively, and that body esteem and social physique anxiety can also play a chained mediating role.

**Conclusion:**

This study reveals the relationship between media internalized pressure and restrained eating behavior, and the important role played by body esteem and social physique anxiety. Future interventions targeting restrained eating should focus on the aspects of body esteem and social physique anxiety.

## Introduction

Restrained eating behavior refers to individuals restricting their food intake for weight loss (Herman and Polivy, 1975; Westenhoefer et al., 2013), and is prevalent among college students (Han and Kahn, 2017). A Chinese app called Mint, which focuses on recording diets, reports that 7.65 million people are involved in restrained eating (Yang et al., 2021). In addition, restrained eating may lead to eating disorders (e.g., bulimia nervosa) and negative psychological emotions, such as anxiety and stress (Stice, 2002; Appleton and McGowan, 2006; Kalkan Ugurlu et al., 2021). Therefore, it is necessary to study the restrained eating behavior of college students and the factors influencing it.

Long-term exposure to the mass media can have a range of harmful effects on college students, such as feeling of isolation (Wright and Pritchard, 2009), anxiety (Garvin and Damson, 2008), and media internalized pressure (Ouyang et al., 2021). Media internalized pressure refers to the pressure that arises after an individual internalizes the ideal body shape which is promoted by the media and mentally compares it with his or her body shape (Stice et al., 2000; Thompson and Stice, 2001; Dittmar et al., 2009). It can relate to body dissatisfaction (Myers and Crowther, 2007), shame (Izydorczyk et al., 2020), stress (Sharp et al., 2014), and even bring about eating problems (Thompson and Heinberg, 1999; Morton et al., 2020). The tripartite influence model of sociocultural theory suggests that the media is one of the initial sources of influence that leads individuals to develop restrained eating behavior (Thompson et al., 1999; Thompson and Stice, 2001). Previous studies have demonstrated that images of slim bodies on television and idealized body size in software can affect individual eating behavior (Mills et al., 2002; Anschutz et al., 2008). This is particularly common among women (Fitzsimmons-Craft et al., 2016) and young adults (Wardle et al., 2006). Austin and Smith (2008) indicated that media internalized pressure can lead individuals to pay more attention to their body shape and create a desire for the ideal body shape, which may lead to restrained eating behavior.

Body esteem may mediate the relationship between media internalized stress and restrained eating behavior. Body esteem is a specific domain of overall self-esteem and refers to an individual's satisfaction with different aspects of the self-body or a positive or negative self-evaluation (Auslander et al., 2012; Lamb et al., 2021). On the one hand, some studies have confirmed that body esteem may relate to restrained eating behavior (Murray et al., 2016). The transdiagnostic theory of eating disorders proposed by Fairburn et al. (2003) suggests that when people are unable to meet the standards set for themselves, they develop negative evaluations of their bodies, which leads to lower body esteem and ultimately leads individuals to develop eating disorders. On the other hand, media internalized pressure may exert an influence on body esteem (Varnes et al., 2015). Flament et al. (2012) demonstrated that both appearance esteem and weight esteem play a mediating role between thin-ideal body shape and restricted eating behavior in female adolescents. When individuals perceive that their body figures are inconsistent with the toned and lean image advocated by the media, this lowers their body esteem and can easily trigger their restrained eating behavior to achieve the ideal figure (Karacan et al., 2014; Gattario et al., 2020).

Furthermore, previous research has demonstrated that social physique anxiety is associated with media internalized stress and restrained eating behavior (Frederick and Morrison, 1998; Ouyang et al., 2021). Social physique anxiety refers to the anxiety that arises from the fear that others will judge one's physique negatively (Hart et al., 1989). Hayes and Ross (1987) suggested that social physique anxiety may lead women to engage in unhealthy eating behaviors due to the pursuit of a thin-ideal body shape. In addition, Sharp et al. (2014) showed that excessive media internalized pressure is a cause of women's body dissatisfaction and social physique anxiety. Individuals who discover a discrepancy between their body shape and the ideal body shape advertised by the media may experience dissatisfaction and anxiety (Christian et al., 2021), and they are prone to more body shape management, such as restrained eating behavior (Thompson et al., 1999).

In addition, body esteem may correlate with social physique anxiety (Gregus et al., 2014). People who are dissatisfied with their physical image may feel anxious when facing others' evaluation of their physical image (Frederick and Morrison, 1996). Miller and Fry (2018) found that body esteem is significantly and negatively associated with social physique anxiety. Atalay and Gençöz (2008) argued that people who have negative perceptions of their body image are more likely to experience higher levels of social physique anxiety. People with high media internalized pressure are more likely to develop lower body esteem (Cordero, 2011) and higher social physique anxiety (Martin, 1999), which can eventually make them avoid showing their body image in public. In the end, they tend to further restrict their eating behaviors and make their image meet social standards (Cox et al., 1997).

**Figure 1:**
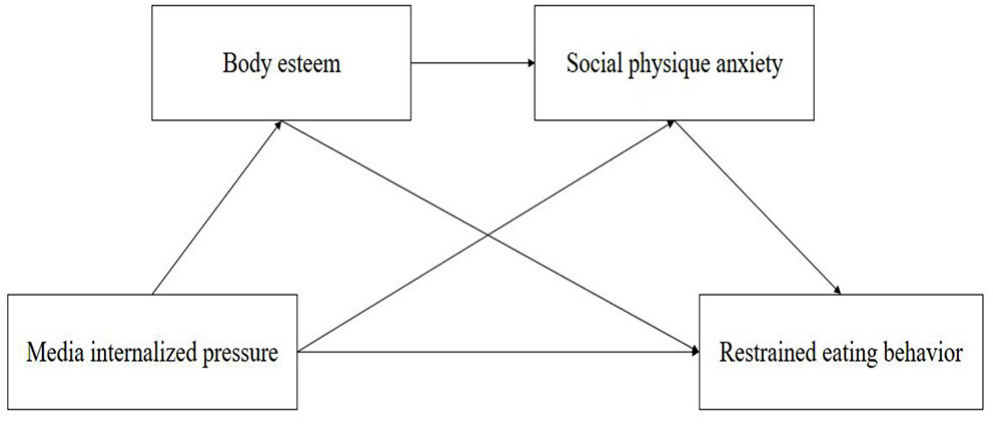
Structural framework.

Our study can further deepen the investigation of factors influencing restrained eating behavior. This study aims to explore the multiple mediating roles of body esteem and social physique anxiety between media internalized pressure and restrained eating behavior among college students. We have proposed the following hypotheses: (1) media internalized pressure is positively associated with college students' restrained eating behavior; (2) the higher the media internalized pressure, the more likely college students are to have lower body esteem and these students' restrained eating behavior will be more frequent, i.e., body esteem plays a mediating role between media internalized pressure and restricted eating behavior; (3) when college students experience greater media internalized pressure, the higher the social physique anxiety they feel and the more frequently restrained eating behavior they experience, i.e., social physique anxiety plays a mediating role between media internalized pressure and restrained eating behavior; and (4) the greater the media internalized pressure on college students, the more likely they may feel lower body esteem and likely to experience higher social physique anxiety, which makes them more likely to engage in restrained eating behavior, i.e., body esteem and social physique anxiety play chained mediating roles between media internalized pressure and restrained eating behavior. The conceptual model is presented in Figure 1.

## Methods

### Participants and Procedures

The participants were all Chinese college students in this study. We collected data from seven universities (i.e., two in North China, one in Central China, three in East China, and one in West China). We distributed the questionnaires through the Questionnaire Star platform (http:www.wjx.cn), which is a software that provides online questionnaire assessment services. We recruited participants in several ways as follows: (1) sent the link of the questionnaire to the class teachers *via* the Internet, who in turn distributed it to their students and (2) recruited participants by posting advertisements on social networks. A total of 1,244 college students participated in the questionnaire, and data from 1,032 participants were eventually included in the data analysis. The survey was anonymous and participants could withdraw from the survey at any time. A total of 212 participants' questionnaires were eliminated, mainly based on invalid responses, such as contradictory responses, and excessive consecutive identical or regular responses. Our study was approved by the Ethics Committee of the School of Public Health at Cheeloo College of Medicine of Shandong University (20190912), and all participants who participated in the survey gave informed consent to the study.

### Measures

#### Demographic Variables

Demographic variables included gender, age, grade, majors, place of residence, and whether the child is an only child. Grades were divided into freshmen, sophomores, juniors, and seniors. Residence included urban and village areas. Majors were classified as science, liberal arts, engineering, and medicine.

#### Sociocultural Attitudes Toward Appearance Questionnaire-3 (SATAQ-3)

This study used the Chinese version (Liu, 2009) of the Sociocultural Attitudes Toward Appearance Questionnaire-3 (SATAQ-3) which was developed by Thompson et al. (2004) to measure media internalized pressure. This scale has 15 items, including a media attention subscale and an internalization subscale. The total score was obtained by adding the scores of the two subscales. This scale is compiled by the 5-point Likert-type scale, with scores of 1–5 meaning “totally disagree” to “totally agree.” Items 6, 10, 11, 12, and 13 reversed scoring questions. The higher the total score, the greater the media internalized pressure they received. The total Cronbach's α coefficient in this study for the scale was 0.864, of which 0.725 for the media attention subscale, and 0.834 for the internalization subscale.

#### Specific Body Esteem Scale (SBES)

The Body Esteem Scale (BES) was designed by Franzoi and Shields (1984). This scale has 35 items, requiring participants to assess various parts and functions of their bodies. In this study, we used the translated and modified Chinese version of BES by He and Zhang (2002), also known as the Specific BES (SBES). This scale includes satisfaction ratings of different body parts and has high reliability and validity. It is compiled by the 5-point Likert-type scale, with scores from 1 to 5 representing “fully unsatisfied” to “fully satisfied,” with higher scores showing higher body esteem. The scale we used showed a Cronbach's α coefficient of 0.969.

#### Social Physique Anxiety Scale (SPAS)

The Social Physique Anxiety Scale (SPAS) was designed by Hart et al. (1989), and we used the Chinese version (Xu, 2007) to gauge the social physique anxiety of college students in this study. This scale consists of 15 items and is compiled by the 5-point Likert-type scale, with scores of 1–5 meaning “not at all” to “complete.” The scores on items l, 3, 7, 11, 13, and 14 reversed in this scale. The higher the total score, the higher the social physique anxiety. Previous studies have demonstrated the applicability of this scale among the Chinese University student population (Liu and Wang, 2019). The scale we used showed a Cronbach's α coefficient of 0.771.

#### Dutch Eating Behavior Questionnaire (DEBQ)

The restrained eating subscale of the Dutch Eating Behavior Questionnaire (DEBQ) developed by Van Strien et al. (1986) was used in this study. There are ten entries, which are primarily used to measure an individual's propensity to eat restrictively. This scale is compiled by the 5-point Likert-type scale and has 10 items. The scores 1, 2, 3, 4, and 5 represent the options “never,” “rarely,” “sometimes,” “often,” and “always,” respectively. There is a higher level of restrained eating behavior with higher scores. The reliability and validity of this scale were good in the Chinese University student population (Li et al., 2018; Ma and Lan, 2021). The Cronbach's α coefficient for this scale was 0.943.

### Statistical Analysis

The SPSS version 24.0 software and its macro program PROCESS version 3.3 (Hayes, 2017) were used to analyze the data. Descriptive statistics, such as frequencies, means, and standard deviations, were used to illustrate the distribution of the variables. Pearson correlation analysis was used to test the relationship between each pair of variables. Model 6 of PROCESS was used for the mediation analysis and to test our hypothesis. The significance levels for the variables were 0.05. The significance of the mediation effect was tested using the Bootstrap method. A Bootstrap sample of 2,000 was set to be drawn in PROCESS, and 95% confidence intervals (CIs) were constructed. The parameter estimates were significant if the 95% CI did not contain 0 (Wen et al., 2004).

## Results

### Common Method Bias Analysis

We used factor analysis to perform the tests of common method bias. The chi-square statistic of Bartlett's test of sphericity was significant (KMO = 0.959, *p* < 0.001). Through the principal component analysis, we extracted a total of nine components with eigenvalues >1. It was found that 24.95% of the variance was explained for the first factor, which was lower than the required standard of 40%. This proves that the questionnaire used in this study has no common method bias (Podsakoff et al., 2003).

### Demographic Variables

We finally analyzed data from 1,032 participants. The mean age of the participants was 20.22 years (SD = 1.277), with an age range of 18–27 years. The final sample contained 439 (42.54%) male students and 593 (57.46%) female students. Table 1 shows the information on demographic variables.

**Table 1:** Demographic data (*N* = 1,032).

**Variables**	**Category**	***N***	**%**
**Gender**
	Male	439	42.54
	Female	593	57.46
**Area**
	Rural	521	50.48
	Town	439	42.52
**Number of children**
	Only one child	521	50.48
	Non-only child	439	42.52
**Grade**
	Freshman	316	30.62
	Sophomore	400	38.76
	Junior	202	19.57
	Senior	114	11.05
**Major**
	Sciences	252	24.42
	Liberal arts	304	29.46
	Engineering	318	30.81
	Medicine	158	15.31

### Correlation Analysis

Table 2 shows the descriptive statistics and correlations between media internalized pressure, body esteem, social physique anxiety, and restrained eating behavior. The bivariate correlation analysis revealed a significant positive relationship between media internalized pressure, social physique anxiety, and restrained eating behavior. In addition, there was a significant negative correlation between body esteem, media internalized pressure, and social physique anxiety.

**Table 2:** Descriptive statistics and correlation analysis for each variable (*n* = 1,032).

**Variables**	***M***	**SD**	**1**	**2**	**3**	**4**
1 Media Internalized Pressure	45.18	10.382	1.000	–	–	–
2 Body Esteem	117.05	26.713	−0.142^**^	1.000	–	–
3 Social Physique Anxiety	44.49	8.796	0.377^**^	−0.333^**^	1.000	–
4 Restrained Eating Behavior	27.18	9.609	0.289^**^	0.083^**^	0.295^**^	1.000

M, Mean; SD, Standard Deviation; **p <0.01.

### Regression Analysis

We performed the regression analysis after standardizing the variables. Table 3 shows the mediated regression coefficients between media internalized pressure, body esteem, social physique anxiety, and restrained eating behavior. The results showed that there was a significant negative correlation between media internalized pressure and body esteem and a significant positive correlation with social physique anxiety. Moreover, there was a significant negative correlation between body esteem and social physique anxiety. Furthermore, media internalized pressure, body esteem, and social physique anxiety can all significantly and positively relate to restrained eating behavior.

**Table 3:** The results of the regression estimate of the chained mediation model.

**Outcome variables**	**Predictors**	**Goodness-of-fit indices**			**Regression coefficient and significance**	
		***R***	***R* ^2^**	***F***	**β**	***t***
BE	MIP	0.263	0.069	7.575^***^	−0.116	−3.751^***^
SPA	MIP	0.477	0.227	27.249^***^	0.339	11.932^***^
	BE				−0.283	−9.904^***^
REB	MIP	0.413	0.171	17.477^***^	0.206	6.551^***^
	BE				0.195	6.311^***^
	SPA				0.288	8.876^***^

***p <0.001. MIP, Media Internalized Pressure; BE, Body Esteem; SPA, Social Physique Anxiety; REB, Restrained Eating Behavior.

### Mediation Analysis

**Figure 2:**
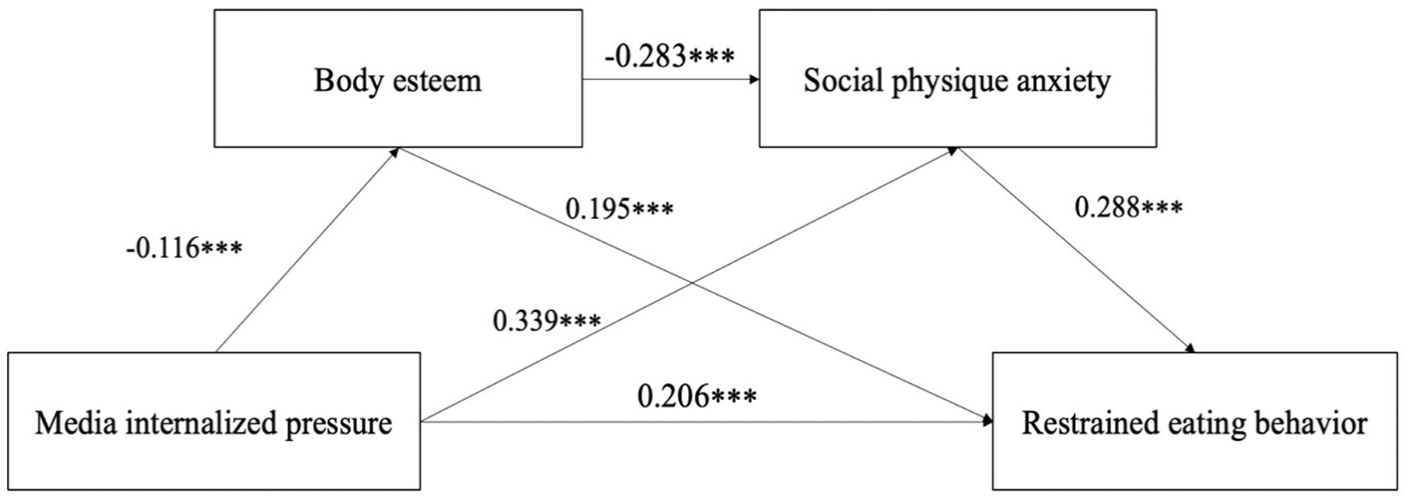
Mediation effect analysis of body esteem and social physique anxiety between media internalized pressure and restrained eating behavior; ***p < 0.001.

The bias-corrected percentile Bootstrap method was used to test all the above-mediated paths, and the selected Bootstrap self-sampling size was 2,000, with 95% CIs calculated. Findings from the mediation analysis are shown in Table 4 and Figure 2. Both the direct and indirect pathways between media internalized pressure and restrained eating behavior were significant. In addition, the separate mediating and chaining effects of body esteem and social physique anxiety between media internalized pressure and restrained eating behavior were also significant.

**Table 4:** Bootstrap analysis of the test for mediating effects (*n* = 1,032).

**Effect types**	**Path**	**Effect**	***SE***	**Bootstrap 95% CI**
Direct effect	MIP → REB	0.206	0.031	0.144–0.268
Indirect effect	MIP → BE → REB	−0.023	0.008	−0.039 to −0.009
	MIP → SPA → REB	0.098	0.016	0.067–0.131
	MIP → BE → SPA → REB	0.010	0.003	0.004–0.016
Total indirect effect		0.085	0.0162	0.054–0.118
Total effect		0.291	0.031	0.231–0.350

*MIP, Media Internalized Pressure; BE, Body Esteem; SPA, Social Physique Anxiety; REB, Restrained Eating Behavior*.

## Discussion

This study investigated the association between media internalized pressure and restrained eating behavior, and the multiple mediating roles of body esteem and social physique anxiety in the relationship between media internalized pressure and restrained eating behavior among college students. We found that (1) there was a significant and positive association between media internalized pressure and restrained eating behavior; (2) body esteem mediated the effect of media internalized pressure on restricted eating behavior; (3) social physique anxiety mediated the effect of media internalized pressure on restricted eating behavior; and (4) body esteem and social physique anxiety play chained mediating roles between media internalized pressure and restrained eating behavior.

Our study found that media internalized pressure was positively related to restrained eating behavior in our college student sample. Similar to our previous studies, Chang et al. (2013) showed that the ideal body shape communicated by mass media leads to media internalized pressure in individuals, which can make them more prone to self-objectification (Slater and Tiggemann, 2015) and thus increase restrained eating behavior (Ashikali and Dittmar, 2010). It highlights the relationship between visual exposure to the media's thin-ideal body shape and restrained eating behavior. This is perhaps because media internalized pressure increases the likelihood of perceived body dissatisfaction, and this appearance pressure, in turn, contributes to restrained eating behavior (Myers and Crowther, 2007; Fuller-Tyszkiewicz et al., 2018).

We found that media internalized pressure can relate to restrained eating behavior through body esteem and social physique anxiety, respectively, which supports our hypotheses 2 and 3. According to the cognitive behavioral model of eating disorders, the thin-ideal body shape promoted by the media increases individuals' misperceptions of their bodies during self-evaluation, which may cause low body esteem and anxiety, and even lead individuals to adopt unhealthy eating behaviors, such as restrained eating behavior (Fairburnm, 1997). Previous studies have also indicated that the internalization pressure of ideal-thin body shape from the media may increase personal body dissatisfaction and decrease body esteem (Hawkins et al., 2004; Vartanian and Dey, 2013). In addition, we found a mediating role for social physique anxiety between media internalized pressure and restricted eating behavior. The mass media's promotion of the ideal body shape can lead individuals to pursue an unrealistically thin-ideal body shape, generating media internalized pressure and dissatisfaction with their body shape, which can lead to social physique anxiety and eventually adopt some restrained eating behavior to reduce this anxiety (Martins et al., 2007; Kertechian and Swami, 2016; Kalyva et al., 2021).

Finally, we found body esteem and social physique anxiety play a chained mediating role between media internalized pressure and restricted eating behavior. This result supports our hypothesis 4. Although the tripartite influence model of the sociocultural theory states that media internalized pressure is one of the causes of restrained eating behavior in individuals (Thompson et al., 1999), this study introduces body esteem and social physique anxiety into this model for the first time and examines the mechanisms by which all three play a role in restrained eating behavior in college students. The media promotion of a thin-ideal body shape leads individuals to evaluate their body shape from the perspective of others, and this inappropriate evaluation tends to lower body esteem and develop social physique anxiety in dissatisfaction with their bodies (Gregus et al., 2014), which leads to thoughts of changing their body shape and eventually results in restrained eating behavior.

This study has important implications. In theoretical aspects, first, we confirmed the relationship between media internalized pressure and restrained eating behavior among college students, extending the tripartite influence model of the sociocultural theory. Second, we applied body esteem and social physique anxiety to explain their association, which helps to understand the underlying mechanisms between these two variables. In practice, we can develop some strategies to avoid restrained eating and other unhealthy behaviors based on these influencing factors. First, social media should stop promoting a single strict aesthetic standard, and instead, should promote healthy beauty and lead people to a healthy lifestyle. Second, we can promote the physical and mental health of college students by promoting correct aesthetic concepts, so that they can be guided to correctly view the ideal body shape and discover their own beauty as disseminated by the media, and thus form correct body perceptions and self-evaluations. Third, we can intervene in mediating variables to reduce restrained eating behavior. Physical activity and restrained eating behavior are the two most commonly used methods of body management, but physical activity rarely produces negative effects (Boutelle et al., 2009; Schur et al., 2010). Encouraging college students to participate in physical activity can also help individuals improve self-confidence and self-esteem, which may reduce restrained eating behavior (Duncan et al., 2009). In addition, mindfulness, a self-regulation mental training method with Buddhist origins, has been shown to be effective in reducing anxiety and depression, and enhancing well-being, which may also help reduce restrained eating behavior (Kristeller and Wolever, 2010).

Despite these positive results, this study also has some limitations. First, this study is a cross-sectional study, so it does not draw a causal relationship between the variables. Future studies could examine the causal relationship between mediating variables and restrained eating behavior or design intervention experiments for mediating variables to test whether restrained eating behavior can be reduced. Second, our survey participants are all college students, and we hope that future research will be extended to include more samples (e.g., adolescents or working adults). Finally, this study adopted an online method of distributing questionnaires; the measurement environment cannot be guaranteed to be consistent and, therefore, may introduce bias. Future studies may consider distributing and collecting questionnaires on-site.

## Conclusion

This study confirms the relationship between media internalized pressure, body esteem, social physique anxiety, and restrained eating behavior. There is a significant and positive association between media internalized pressure and restrained eating behavior among college students. Moreover, media internalized pressure can also increase the risk of restrained eating behavior through the parallel mediating effects of body esteem and social physique anxiety, as well as through the sequential mediating effects of body esteem and social physique anxiety.

## Data Availability Statement

The original contributions presented in the study are included in the article/supplementary material, further inquiries can be directed to the corresponding author/s.

## Ethics Statement

The studies involving human participants were reviewed and approved by Ethics Committee of the School of Public Health at Cheeloo College of Medicine of Shandong University (20190912). The patients/participants provided their written informed consent to participate in this study.

## Author Contributions

TF and SX completed the writing of the introduction and discussion. JY completed the writing of the methods. TF completed the writing of results, data coding, cleaning, and the first draft of the manuscript. JW and GS completed the writing review and editing. All authors contributed to the article and approved the submitted version.

## Funding

This research was supported by the Social Science Fund of Shandong Province, China (No. 18DTYJ02), the Teaching Research Project of Shandong University (No. XYJG2020025), and the Young Scholars Program of Shandong University.

## Conflict of Interest

The authors declare that the research was conducted in the absence of any commercial or financial relationships that could be construed as a potential conflict of interest.

## Publisher's Note

All claims expressed in this article are solely those of the authors and do not necessarily represent those of their affiliated organizations, or those of the publisher, the editors and the reviewers. Any product that may be evaluated in this article, or claim that may be made by its manufacturer, is not guaranteed or endorsed by the publisher.

## References

[B1] Anschutz D. J. Van Strien T. Engels R. C. (2008). Exposure to slim images in mass media: television commercials as reminders of restriction in restrained eaters. Health Psychol. 27, 401. 10.1037/0278-6133.27.4.401 18642997

[B2] Appleton K. M. McGowan L. (2006). The relationship between restrained eating and poor psychological health is moderated by pleasure normally associated with eating. Eat. Behav. 7, 342–347. 10.1016/j.eatbeh.2005.11.008 17056410

[B3] Ashikali E. M. Dittmar H. (2010). Body image and restrained eating in blind and sighted women: a preliminary study. Body Image 7, 172–175. 10.1016/j.bodyim.2010.01.002 20185377

[B4] Atalay A. A. Gençöz T. (2008). Critical factors of social physique anxiety: exercising and body image satisfaction. Behav. Change 25, 178–188. 10.1375/bech.25.3.178

[B5] Auslander B. A. Baker J. Short M. B. (2012). The connection between young women's body esteem and sexual assertiveness. J. Pediatr. Adolesc. Gynecol. 25, 127–130. 10.1016/j.jpag.2011.11.008 22260892

[B6] Austin J. L. Smith J. E. (2008). Thin ideal internalization in Mexican girls: a test of the sociocultural model of eating disorders. Int. J. Eat. Disord. 41, 448–457. 10.1002/eat.20529 18433029

[B7] Boutelle K. N. Libbey H. Neumark-Sztainer D. Story M. (2009). Weight control strategies of overweight adolescents who successfully lost weight. J. Am. Diet. Assoc. 109, 2029–2035. 10.1016/j.jada.2009.09.012 19942020

[B8] Chang F. C. Lee C. M. Chen P. H. Chiu C. H. Pan Y. C. Huang T. F. (2013). Association of thin-ideal media exposure, body dissatisfaction and disordered eating behaviors among adolescents in Taiwan. Eat. Behav. 14, 382–385. 10.1016/j.eatbeh.2013.05.002 23910785

[B9] Christian C. B. Ngo B. K. Brosof L. C. Levinson C. A. (2021). Social appearance anxiety moderates the relationship between thin-ideal internalization and eating disorder symptoms cross-sectionally and prospectively in adolescent girls. Eat. Weight Disord. 26, 2065–2070. 10.1007/s40519-020-01050-y 33106938

[B10] Cordero E. D (2011). Self-esteem, social support, collectivism, and the thin-ideal in Latina undergraduates. Body Image 8, 82–85. 10.1016/j.bodyim.2010.11.006 21147052 PMC3063392

[B11] Cox L. M. Lantz C. D. Mayhew J. L. (1997). The role of social physique anxiety and other variables in predicting eating behaviors in college students. Int. J. Sport Nutr. 7, 310–317. 10.1123/ijsn.7.4.310 9407257

[B12] Dittmar H. Halliwell E. Stirling E. (2009). Understanding the impact of thin media models on women's body-focused affect: the roles of thin-ideal internalization and weight-related self-discrepancy activation in experimental exposure effects. J. Soc. Clin. Psychol. 28, 43–72. 10.1521/jscp.2009.28.1.43

[B13] Duncan M. J. Al-Nakeeb Y. Nevill A. M. (2009). Effects of a 6-week circuit training intervention on body esteem and body mass index in British primary school children. Body Image 6, 216–220. 10.1016/j.bodyim.2009.04.003 19447693

[B14] Fairburn C. G. Cooper Z. Shafran R. (2003). Cognitive behaviour therapy for eating disorders: a “transdiagnostic” theory and treatment. Behav Res Ther. 41, 509–528. 10.1016/S0005-7967(02)00088-8 12711261

[B15] Fairburnm C. G (1997). “Eating disorders,” in Science and Practice of Cognitive Behaviour Therapy, eds D. M. Clark, and C. G. Fairburn (America: Oxford University Press), p. 209–241.

[B16] Fitzsimmons-Craft E. E. Bardone-Cone A. M. Crosby R. D. Engel S. G. Wonderlich S. A. Bulik C. M. (2016). Mediators of the relationship between thin-ideal internalization and body dissatisfaction in the natural environment. Body Image, 18, 11300122. 10.1016/j.bodyim.2016.06.006 PMC5012939 27391791

[B17] Flament M. F. Hill E. M. Buchholz A. Henderson K. Tasca G. A. Goldfield G. (2012). Internalization of the thin and muscular body ideal and disordered eating in adolescence: the mediation effects of body esteem. Body Image 9, 68–75. 10.1016/j.bodyim.2011.07.007 21889429

[B18] Franzoi S. L. Shields S. A. (1984). The body esteem scale: multidimensional structure and sex differences in a college population. J. Pers. Assess. 48, 173–178. 10.1207/s15327752jpa4802_12 6726603

[B19] Frederick C. M. Morrison C. S. (1996). Social physique anxiety: personality constructs, motivations, exercise attitudes, and behaviors. Percept. Motor Skills. 82, 963–972. 10.2466/pms.1996.82.3.963 8774039

[B20] Frederick C. M. Morrison C. S. (1998). A mediational model of social physique anxiety and eating disordered behaviors. Percept. Motor Skills 86, 139–145. 10.2466/pms.1998.86.1.139 9530723

[B21] Fuller-Tyszkiewicz M. Richardson B. Lewis V. Smyth J. Krug I. (2018). Do women with greater trait body dissatisfaction experience body dissatisfaction states differently? An experience sampling study. Body Image 25, 1–8. 10.1016/j.bodyim.2018.01.004 29413880

[B22] Garvin A. W. Damson C. (2008). The effects of idealized fitness images on anxiety, depression and global mood states in college age males and females. J. Health Psychol. 13, 433–437. 10.1177/1359105307088146 18420776

[B23] Gattario K. H. Frisén A. Teall T. L. Piran N. (2020). Embodiment: cultural and gender differences and associations with life satisfaction. Body Image 35, 1–10. 10.1016/j.bodyim.2020.07.005 32877841

[B24] Gregus S. J. Rummell C. M. Rankin T. J. Levant R. F. (2014). Women's experiences of sexual attention: a cross-sectional study of US University students. Int. J. Sex. Health 26, 239–257. 10.1080/19317611.2014.885922

[B25] Han S. Kahn J. H. (2017). Attachment, emotion regulation difficulties, and disordered eating among college women and men. Couns. Psychol. 45, 1066–1090. 10.1177/0011000017744884

[B26] Hart E. A. Leary M. R. Rejeski W. J. (1989). Tie measurement of social physique anxiety. J. Sport Exerc. Psychol. 11, 94–104. 10.1123/jsep.11.1.94

[B27] Hawkins N. Richards P. S. Granley H. M. Stein D. M. (2004). The impact of exposure to the thin-ideal media image on women. Eat. Disord. 12, 35–50. 10.1080/10640260490267751 16864303

[B28] Hayes A. F (2017). Introduction to Mediation, Moderation, and Conditional Process Analysis: a Regression-Based Approach. Guilford Publications.

[B29] Hayes D. Ross C. E. (1987). Concern with appearance, health beliefs, and eating habits. J. Health Soc Behav. 28, 120–130. 10.2307/2137126 3611701

[B30] He L. Tension L. (2002). The relationship between abstraction and its specific body self-esteem evaluation style and life satisfaction[J]. J. Beijing Univ. Phys. Educ. Sports. 320–323+330. 10.19582/j.cnki.11-3785/g8.2002.03.012

[B31] Herman C. P. Polivy J. (1975). Anxiety, restraint, and eating behavior. J. Abnorm. Psychol. 84, 666. 10.1037/0021-843X.84.6.666 1194527

[B32] Izydorczyk B. Sitnik-Warchulska K. Lizińczyk S. Lipowska M. (2020). Socio-cultural standards promoted by the mass media as predictors of restrictive and bulimic behavior. Front. Psychiatry 11, 506. 10.3389/fpsyt.2020.00506 32581880 PMC7283604

[B33] Kalkan Ugurlu Y. Mataraci Degirmenci D. Durgun H. Gök Ugur H. (2021). The examination of the relationship between nursing students' depression, anxiety and stress levels and restrictive, emotional, and external eating behaviors in COVID-19 social isolation process. Perspect. Psychiat. Care 57, 507–516. 10.1111/ppc.12703 33270226 PMC7753737

[B34] Kalyva S. Yannakoulia M. Koutsouba M. Venetsanou F. (2021). Disturbed eating attitudes, social physique anxiety, and perceived pressure for thin body in professional dancers. Res. Dance Educ. 1–12. 10.1080/14647893.2021.1940124

[B35] Karacan E. Caglar G. S. Gürsoy A. Y. Yilmaz M. B. (2014). Body satisfaction and eating attitudes among girls and young women with and without polycystic ovary syndrome. J. Pediat. Adolesc. Gynecol. 27, 72–77. 10.1016/j.jpag.2013.08.003 24602301

[B36] Kertechian S. K. Swami V. (2016). The hijab as a protective factor for body image and disordered eating: a replication in French Muslim women. Ment. Health Relig. Cult. 19, 1056–1068. 10.1080/13674676.2017.1312322

[B37] Kristeller J. L. Wolever R. Q. (2010). Mindfulness-based eating awareness training for treating binge eating disorder: the conceptual foundation. Eat Disord. 19, 49–61. 10.1080/10640266.2011.533605 21181579

[B38] Lamb M. Buchholz A. Gunnell K. E. Valois D. D. Obeid N. Henderson K.. (2021). Examining the bidirectional association between body esteem and body mass index during adolescence. J. Dev. Behav. Pediat. 42, 631–636. 10.1097/DBP.0000000000000945 33908378

[B39] Li Y. N. Liu Y. Bao J. (2018). Application of the Chinese version of the Dutch Eating Behavior Questionnaire to a population of Chinese University students. Chin. J. Clin. Psychol. 277–281+326.

[B40] Liu D (2009). A Study on the Influence of Mass Media and Peers on College Students' Body Imagery. Xiamen: Xiamen University.

[B41] Liu J. Wang X. (2019). The effect of dance on anxiety and physical and mental health of female college students. J. Guangzhou Inst. Phys. Educ. 107–110. 10.13830/j.cnki.cn44-1129/g8.2019.05.024

[B42] Ma Y. Lan Y. (2021). The effect of ideal beauty internalization on college students'restrictive diets: a chain mediated effect analysis. Chin. J. Health Psychol. 30:1–7. 10.13342/j.cnki.cjhp.2022.03.024

[B43] Martin J. J (1999). Predictors of social physique anxiety in adolescent swimmers with physical disabilities. Adapt. Phys. Activ. Q. 16, 75–85. 10.1123/apaq.16.1.75

[B44] Martins Y. Tiggemann M. Kirkbride A. (2007). Those speedos become them: the role of self-objectification in gay and heterosexual men's body image. Pers. Soc. Psychol. Bull. 33, 634–647. 10.1177/0146167206297403 17440202

[B45] Miller S. Fry M. (2018). Relationship between motivational climate to body esteem and social physique anxiety within college physical activity classes. J. Clin. Sport Psychol. 12, 525–543. 10.1123/jcsp.2018-0005

[B46] Mills J. S. Polivy J. Herman C. P. Tiggemann M. (2002). Effects of exposure to thin media images: evidence of self-enhancement among restrained eaters. Pers. Soc. Psychol. Bull. 28, 1687–1699. 10.1177/014616702237650

[B47] Morton C. Mooney T. A. Lozano L. L. Adams E. A. Makriyianis H. M. Liss M. (2020). Psychological inflexibility moderates the relationship between thin-ideal internalization and disordered eating. Eat. Behav. 36, 101345. 10.1016/j.eatbeh.2019.101345 31760368

[B48] Murray M. Maras D. Goldfield G. S. (2016). Excessive time on social networking sites and disordered eating behaviors among undergraduate students: appearance and weight esteem as mediating pathways. Cyberpsychol. Behav. Soc. Netw. 19, 709–715. 10.1089/cyber.2016.0384 27925798

[B49] Myers T. A. Crowther J. H. (2007). Sociocultural pressures, thin-ideal internalization, self-objectification, and body dissatisfaction: could feminist beliefs be a moderating factor?. Body Image 4, 296–308. 10.1016/j.bodyim.2007.04.001 18089276

[B50] Ouyang Y. Luo J. Teng J. Zhang T. Wang K. Li J. (2021). Research on the Influence of Media Internalized Pressure on College Students' Sports Participation—Chained Intermediary Analysis of Social Physique Anxiety and Weight Control Self-Efficacy. Front. Psychol. 12, 654690. 10.3389/fpsyg.2021.654690 34054659 PMC8149783

[B51] Podsakoff P. M. MacKenzie S. B. Lee J. Y. Podsakoff N. P. (2003). Common method biases in behavioral research: a critical review of the literature and recommended remedies. J. Appl. Psychol. 88, 879. 10.1037/0021-9010.88.5.879 14516251

[B52] Schur E. A. Heckbert S. R. Goldberg J. H. (2010). The association of restrained eating with weight change over time in a community-based sample of twins. Obesity 18, 1146–1152. 10.1038/oby.2009.506 20111024 PMC3954714

[B53] Sharp G. Tiggemann M. Mattiske J. (2014). The role of media and peer influences in Australian women's attitudes towards cosmetic surgery. Body Image 11, 482–487. 10.1016/j.bodyim.2014.07.009 25129686

[B54] Slater A. Tiggemann M. (2015). Media exposure, extracurricular activities, and appearance-related comments as predictors of female adolescents' self-objectification. Psychol. Women Q. 39, 375–389. 10.1177/0361684314554606

[B55] Stice E (2002). Risk and maintenance factors for eating pathology: a meta-analytic review. Psychol. Bull. 128, 825. 10.1037/0033-2909.128.5.825 12206196

[B56] Stice E. Mazotti L. Weibel D. Agras W. S. (2000). Dissonance prevention program decreases thin-ideal internalization, body dissatisfaction, dieting, negative affect, and bulimic symptoms: a preliminary experiment. Int. J. Eat. Disord. 27, 206–217. 10.1002/(SICI)1098-108X(200003)27:2<206::AID-EAT9>3.0.CO;2-D 10657894

[B57] Thompson J. K. Heinberg L. J. (1999). The media's influence on body image disturbance and eating disorders: we've reviled them, now can we rehabilitate them?. J. Soc. Issues 55, 339–353. 10.1111/0022-4537.00119

[B58] Thompson J. K. Heinberg L. J. Altabe M. Tantleff-Dunn S. (1999). Exacting Beauty: Theory, Assessment, and Treatment of Body Image Disturbance. American Psychological Association.

[B59] Thompson J. K. Stice E. (2001). Thin-ideal internalization: mounting evidence for a new risk factor for body-image disturbance and eating pathology. Curr. Dir. Psychol. Sci. 10, 181–183. 10.1111/1467-8721.00144

[B60] Thompson J. K. Van Den Berg P. Roehrig M. Guarda A. S. Heinberg L. J. (2004). The sociocultural attitudes towards appearance scale-3 (SATAQ-3): development and validation. Int. J. Eat. Disord. 35, 293–304. 10.1002/eat.10257 15048945

[B61] Van Strien T. Frijters J. E. Bergers G. P. Defares P. B. (1986). The Dutch Eating Behavior Questionnaire (DEBQ) for assessment of restrained, emotional, and external eating behavior. Int. J. Eat. Disord. 5, 295–315. 10.1002/1098-108X(198602)5:2<295::AID-EAT2260050209>3.0.CO;2-T

[B62] Varnes J. R. Stellefson M. L. Miller M. D. Janelle C. M. Dodd V. Pigg R. M. (2015). Body esteem and self-objectification among collegiate female athletes: does societal objectification make a difference?. Psychol. Women Q. 39, 95–108. 10.1177/0361684314531097

[B63] Vartanian L. R. Dey S. (2013). Self-concept clarity, thin-ideal internalization, and appearance-related social comparison as predictors of body dissatisfaction. Body Image 10, 495–500. 10.1016/j.bodyim.2013.05.004 23809858

[B64] Wardle J. Haase A. M. Steptoe A. (2006). Body image and weight control in young adults: international comparisons in University students from 22 countries. Int. J. Obesity 30, 644–651. 10.1038/sj.ijo.0803050 16151414

[B65] Wen Z. Zhang L. Hou J. Liu H. (2004). Mediated effects testing procedures and their applications. J. Psychol. 05, 614–620.

[B66] Westenhoefer J. Engel D. Holst C. Lorenz J. Peacock M. Stubbs J.. (2013). Cognitive and weight-related correlates of flexible and rigid restrained eating behaviour. Eat. Behav. 14, 69–72. 10.1016/j.eatbeh.2012.10.015 23265405

[B67] Wright A. Pritchard M. E. (2009). An examination of the relation of gender, mass media influence, and loneliness to disordered eating among college students. Eat. Weight Disord. 14, e144–e147. 10.1007/BF03327813 19934629

[B68] Xu X (2007). A Study on the Measurement of Social Physical Anxiety and its Relationship With Physical Activity. Beijing Sport University Press.

[B69] Yang L. Wang J-N. Li Q. Zhao S-Q. Jin T-L. (2021). The effect of media image internalization on college students' restricted eating behavior: a mediated model with moderation[J]. Psychological Science. 44:162–168. 10.16719/j.cnki.671-6981.20210123

